# Diverse Vaginal Microbiomes in Reproductive-Age Women with Vulvovaginal Candidiasis

**DOI:** 10.1371/journal.pone.0079812

**Published:** 2013-11-12

**Authors:** Mu-Biao Liu, Su-Rong Xu, Yan He, Guan-Hua Deng, Hua-Fang Sheng, Xue-Mei Huang, Cai-Yan Ouyang, Hong-Wei Zhou

**Affiliations:** 1 Department of Obstetrics and Gynecology, Zhujiang Hospital, Southern Medical University, Guangzhou, Guangdong, China; 2 Microbial Ecology Lab, Department of Environmental Health, School of Public Health and Tropical Medicine, Southern Medical University, Guangzhou, Guangdong, China; Institute for Genome Sciences, University of Maryland School of Medicine, United States of America

## Abstract

Vulvovaginal candidiasis (VVC) is one of the most prevalent vaginal infectious diseases, and there are controversial reports regarding the diversity of the associated vaginal microbiota. We determined the vaginal microbial community in patients with VVC, bacterial vaginosis (BV), and mixed infection of VVC and BV using Illumina sequencing of 16S rRNA tags. Our results revealed for the first time the highly variable patterns of the vaginal microbiome from VVC patients. In general, the alpha-diversity results of species richness and evenness showed the following order: normal control < VVC only < mixed BV and VVC infection < BV only. The beta-diversity comparison of community structures also showed an intermediate composition of VVC between the control and BV samples. A detailed comparison showed that, although the control and BV communities had typical patterns, the vaginal microbiota of VVC is complex. The mixed BV and VVC infection group showed a unique pattern, with a relatively higher abundance of *Lactobacillus* than the BV group and higher abundance of *Prevotella*, *Gardnerella*, and *Atopobium* than the normal control. In contrast, the VVC-only group could not be described by any single profile, ranging from a community structure similar to the normal control (predominated with *Lactobacillus*) to BV-like community structures (abundant with *Gardnerella* and *Atopobium*). Treatment of VVC resulted in inconsistent changes of the vaginal microbiota, with four BV/VVC samples recovering to a higher *Lactobacillus* level, whereas many VVC-only patients did not. These results will be useful for future studies on the role of vaginal microbiota in VVC and related infectious diseases.

## Introduction

The vaginal microbiota comprises a community of microbes with moderate diversity and plays a mutualistic role in the maintenance of vaginal health. Studies have suggested that disruption of the microbial composition can lead to increased susceptibility to various infectious diseases and increased adverse pregnancy outcomes [1]–[3]. The recent development of microbial community determination using next-generation sequencing (NGS) techniques has significantly improved the efficiency of studying vaginal microbiota [2], [4], allowing the high-throughput analysis of hundreds to thousands of samples with detailed taxonomic and abundance information regarding the microbes present. These improvements provide a better understanding of the normal vaginal microbiota and their longitudinal changes in both healthy women and those with bacterial vaginiasis (BV) [4]–[7].

Vulvovaginal candidiasis (VVC) is defined as symptoms of inflammation and an overgrowth of *Candida* spp., particularly *C. albicans*, without other infectious etiologies [8], [9]. VVC is one of the most common types of infectious vaginitis, secondary only to BV as the reason that women seek gynecological care. In a recent survey, up to 40% of women with vaginal complaints in primary care settings were diagnosed as having VVC [9], [10]. Approximately 75% of women experience at least one episode of VVC during their lives, most commonly between the age of 20 to 40 years old [8]. However, an estimated 5% of women with VVC experience recurrent VVC, which is defined as four or more distinct episodes in a single year [9], [11]. Although there have been many studies regarding the host immunity and pathogenesis of *Candida* spp, little attention has been given to the vaginal microbiota, one of the most important aspects of the vaginal environment [10].

The role of vaginal microbiota in VVC is controversial in the literature. VVC is a common side effect of BV treatment with antibiotics, indicating that the vaginal microbiota might be related to the colonization of yeast [12]. There have been reports regarding the association between VVC and intermediate flora patterns that result in an altered vaginal bacterial community [13], [14]. However, a comparison of the *Lactobacillus* species cultured from the vaginal secretions of women with or without VVC showed no significant differences [15]. Moreover, McClelland et al. reported that vaginal *Lactobacillus* colonization was associated with a ≥4-fold increase in the likelihood of symptomatic VVC [16]. Recently, Zhou et al. compared vaginal bacterial communities using the terminal restriction fragment length polymorphism (T-RFLP) technique; however, their results showed no altered or unusual bacterial community in women with VVC, and the authors suggested that commensal vaginal bacterial species might be incapable of preventing VVC [11]. To date, all the reports on the vaginal microbiome of VVC patients have been obtained using traditional cultivation or molecular fingerprinting methods, whereas there are no reports on the vaginal microbiome in VVC using NGS techniques [11]. In the present study, we used the barcoded Illumina paired-end sequencing (BIPES) technique [17] to determine the vaginal bacterial community structure of reproductive-age women with VVC in Guangzhou, China. Our study is the first report on the vaginal microbiota of VVC using NGS methods, which revealed diverse microbial community patterns of the disease.

## Materials and Methods

### Ethical statement

The study was approved by the Ethical Committee of Southern Medical University, and all participants provided informed written consent.

### Subject selection and sample collection

A total of 226 vaginal swabs were collected from 95 participants in the Department of Gynecology of the Second Affiliated Hospital of Southern Medical University in China between June and September 2012. The average age of the participants was 31.7 years old. 30 of the subjects were healthy, 39 were infected with VVC, 16 were infected with BV/VVC and 10 were infected with BV. 14 follow-up subjects were infected with VVC, 4 follow-up subjects recovered from the BV/VVC infection, and 1 follow-up subject was infected with BV. From each subject, we took two swabs, one from the vaginal fornix and the other one from the lower vagina (lower third of vagina). Fresh samples were evaluated for pH using pH-testing strips and then placed in a −70°C refrigerator until DNA extraction. Each individual underwent routine gynecology examination by two gynecologists. VVC is diagnosed via the microscopic detection of dense numbers of yeast cells on a vaginal smear and by physical examination and the presence of a white, mucous-like yeast discharge. BV status was assessed using Amsel's clinical criteria [18] for all subjects and was confirmed using Gram-stain criteria (Nugent scores)[19]. The participants who met three or more of the following criteria were clinically diagnosed with BV: homogenous vaginal discharge, >20% clue cells on wet mount, elevated vaginal discharge pH (≥4.5), and the release of a fishy amine odor upon the addition of 10% potassium hydroxide solution to vaginal fluid (“whiff”test). Then, based on the criteria for BV assessment developed by Nugent et al., participants with a Gram-stain score ≥7 were finally confirmed as having BV. Participants who met the criteria for both VVC and BV were diagnosed with a mixed infection of VVC with BV. Participants without any of these conditions were defined as healthy controls. Participants with any of the following criteria was excluded: <18 years of age, pregnancy, diabetes mellitus, use of antibiotics or vaginal antimicrobials (orally or by topical application to the vulvar/vaginal area) in the previous month, menstruation, menoxenia, the presence of an intrauterine device, known active co-infection with *Chlamydia*, *Neisseria gonorrhoeae*, or *Trichomonas vaginalis*, clinically apparent herpes simplex infection, or diagnosed HPV, HSV- 2, or HIV infection.

### Total bacterial genomic DNA extraction

The bacterial DNA was extracted from the vaginal swabs using the DNA MAGNETIC SAND EXTRACT Kit (BioEAsy, China) according to manufacturer's instructions. The bacterial cells retrieved on swabs were submerged in 250 µl of TNCa buffer and vigorously agitated to dislodge the cells. A total of 20 µl of a proteinase K solution (20 mg/ml) was added and blended and then digested at 56°C for approximately 15 min. Two hundred microliters of the lysis-binding buffer provided in the kit was added and then 200 µl of absolute ethyl alcohol and 40 µl of magnetic beads were added and agitated for 20 s. The samples were left to stand at room temperature for 10 min and were agitated whirled every 2 min. The mixtures were left on a magnetic shelf for 20 s to settle, and the supernatants were discarded. Then, 500 µl of W1 wash buffer was added, the mixture was agitated whirled for 15 s and then placed on a magnetic shelf for 20 s to settle, and the supernatant was discarded. The precipitate was washed with 700 µl of W2 wash buffer. The solution was then unwrapped and kept at 56°C for 7 min. One hundred microliters of elution buffer was added, and the solution was agitated for 15 s. The sample tube was immersed in a 56°C constant temperature bath for 7 min, then removed and agitated for 15 s, placed on a magnetic shelf for 20 s to extract the supernatant, and stored at −20°C before PCR.

### PCR amplification

We used the barcoded 967F (CNACGCGAAGAACCTTANC) and 1046R (CGACAGCCATGCANCACCT) primers to amplify the bacterial 16S rRNA V6 fragments [17]. The PCR cycle conditions were as follows: an initial denaturation at 94°C for 2 min, 24 cycles of 94°C for 30 s, 57°C for 30 s, and 72°C for 30 s, and a final extension at 72°C for 5 min. Each 25-µl reaction consisted of 2.5 µl of Takara 10× Ex Taq Buffer (Mg^2+^ free), 2 µl of dNTP mix (2.5 mM each), 1.5 µl of Mg^2+^ (25 mM each), 0.25 µl of Takara Ex Taq DNA polymerase (2.5 units), 1 µl of template DNA, 0.5 µl of 10 µM barcoded primer 967F, 0.5 µl of 10 µM primer 1406R, and 16.75 µl of ddH2O. Equimolar amplicon suspensions were combined and subjected to paired-end sequencing on anIllumina HiSeq 2000 sequencer at Beijing Genomic Institute.

### Data analysis

The raw sequences were processed using the BIPES pipeline [17], with no errors in the primer regions allowed and only one mismatch allowed in the 40–70 bp region during the overlap step. The variable tags (overlapping lengths minus primers and barcodes) that were shorter than 50 bp, longer than 90 bp or containing ambiguous bases (N) were also removed. All of the qualified reads were then separated according to their barcodes and screened for chimeras using UCHIME[20].

We applied a two-stage-clustering (TSC) algorithm to cluster tags into operational taxonomy units (OTUs) [21]. The cutoff parameter was set to 3, meaning that tags with frequencies of 3 or greater were clustered using a stringent hierarchical clustering algorithm with the Needleman-Wunsch (NW) distance, and tags that occurred 1 or 2 times were clustered using a greedy heuristic algorithm with the NW distance. The clustering distance was 0.03.

The taxonomical assignment of OTUs was performed using the Global Alignment for Sequence Taxonomy (GAST) method, which had good performance for assigning the V6 tag [22]. From each OTU, the sequence with the highest frequency was used as the representative. Diversity index and PCoA analysis using the UniFrac distance were performed with QIIME [23]. Statistical analysis for pH, relative abundance of genera and diversity indices and estimators were performed using SPSS. Differentially abundant features were determined using LEfSe [24]. Datasets were deposited in Sequence Read Archive with accession numbers from ERS348892 to ERS349115 under study PRJEB4606.

## Results

### Sequencing results

A total of 1,251,524 tags were determined from the 226 samples examined. After filtering the low-quality reads and removing chimeras, we obtained 1,026,382 high-quality reads, with an average number of 4,542 reads per sample. All of the sequences were clustered into operational taxonomic units (OTUs) using TSC, and representative sequences from each OTU were used for the taxonomic assignment with GAST, a pipeline with good performance for analyzing V6 tags [22]. We sampled two swabs for each individual: one from the vaginal fornix and the other from the lower vagina. According to our analyses, both of the swabs from the same subject normally showed similar microbiome community structures compared to the swabs from other subjects (grouped together using PCoA and UPGMA clustering and showing similar constituents at all taxonomic levels), thus indicating good microbiome reproducibility for the two sampling sites. Therefore, we pooled the sequences from paired samples in the ensuing analysis. Good's coverage values for all the samples were from 0.980 to 0.998, indicating that our sequencing depth was sufficient to represent the majority of the microbiota in the vaginal environment.

### Microbiome diversity comparison

**Figure 1:**
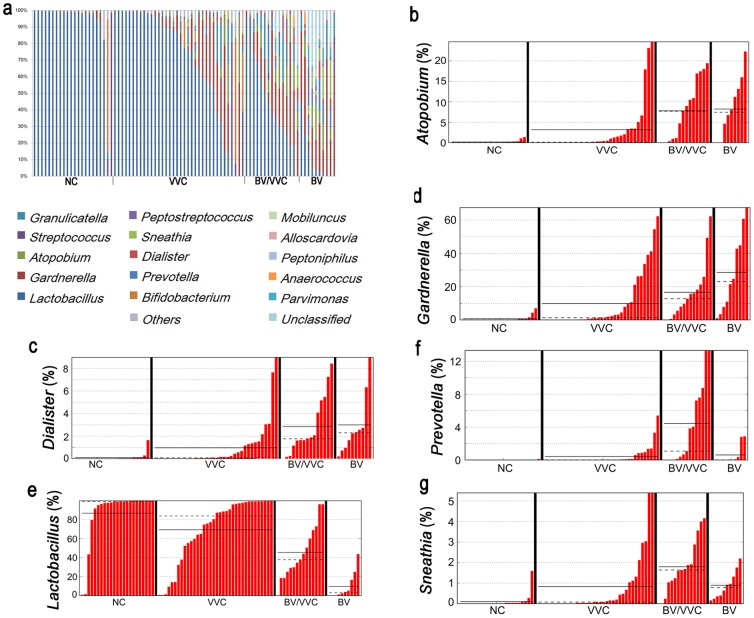
Genus-level distribution of the vaginal microbiota determined in the present study. (a) Relative abundance of NC, VVC, BV/VVC, and BV. (b–h) Percentage of specific genera in NC, VVC, BV/VVC, and BV. (b) *Atopobium*; (c) *Dialister*; (d) *Gardnerella*; (e) *Lactobacillus*; (f) *Prevotella*; (g) *Sneathia*.

For purposes of comparison, we recruited 30 healthy women without any symptoms of vaginitis and 10 BV patients. We further divided the VVC patients into two groups: those with a mixed occurrence of BV and VVC (named BV/VVC in the present study) and those with VVC only (named VVC). The genus level distribution (Fig. 1) clearly shows that the vaginal microbiota of the healthy group was normally dominated by the *Lactobacillus* genus, which is consistent with the common perception of a normal vaginal bacterial community structure [1], [3]. Additionally, we observed two individuals from the normal control (NC) group with vaginal microbiomes dominated by *Bifidobacterium* or *Streptococcus*, both of which have been reported to be temporarily dominant in healthy women without any symptoms [7], [25]. In comparison, the BV patients showed much higher diversity, with a relatively low abundance of *Lactobacillus* and a high abundance of BV-related bacteria, such as *Gardnerella*, *Atopobium*, *Dialister*, *Sneathia*, *Mobiluncus*, and *Prevotella* (Fig. 1); these genera are typical of the vaginal bacterial communities of BV patients determined using both traditional and advanced next-generation sequencing methods [5], [26]–[28]. The consistency of our results for the NC and BV groups with those from the literature confirms the accuracy of our pipeline, and these two groups, NC and BV, were used as references for the following analyses.

**Figure 2:**
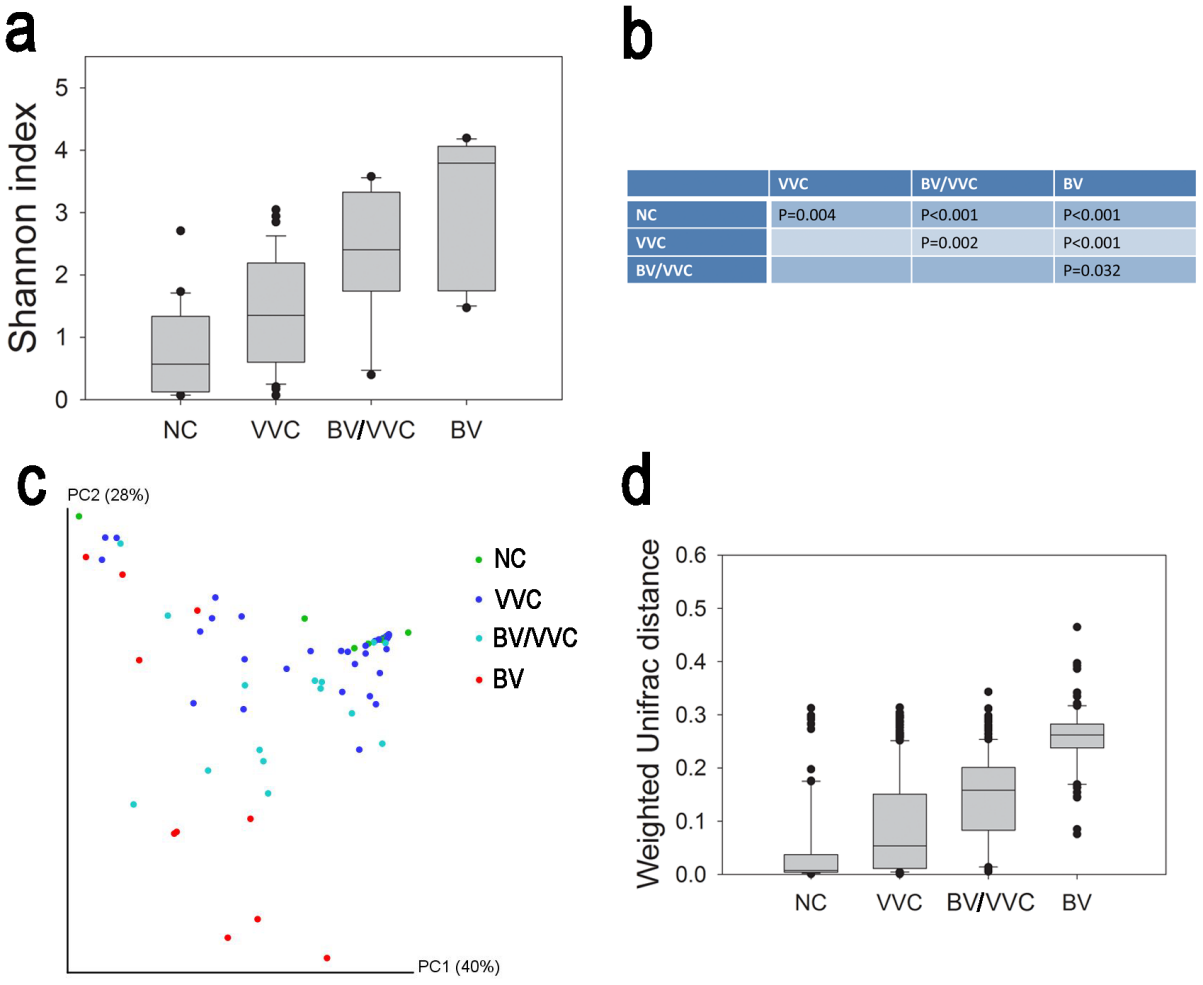
Alpha- and beta-diversity comparison. (a) Comparison of the Shannon index of NC, VVC, BV/VVC, and BV using a one-way ANOVA (p<0.05). (b) Pair-wise comparison of the Shannon index using Dunn's test. (c) PCoA analysis with weighted UniFrac distance. (d) Comparison of the weighted UniFrac distances of each group against NC. The statistical analysis was implemented using one-way ANOVA by ranks (normality test failed, p<0.05) and Dunn's test for pairwise comparisons (p<0.05 for all pair-wise comparisons).

Although the vaginal microbial communities of normal women and BV patients generally have typical patterns, the profiles of vaginal microbiota in VVC patients are rather complicated. According to the Shannon diversity index (Fig. 2), the BV and the two VVC groups (BV/VVC and VVC) showed significantly increased diversity in comparison to the NC group (one-way ANOVA, p<0.05). The order of the four groups increased NC < VVC < BV/VVC < BV, with each pair of these four groups showing a significant difference (one-way-ANOVA, p<0.05). These results indicate that the species richness and evenness of the vaginal microbiome in VVC patients increased and were intermediate between the healthy and BV status.

The beta-diversity comparison of the four groups showed a similar trend with that of the alpha-diversity results. The PCoA analysis with UniFrac distance show that the NC and BV samples were visually separated, whereas the VVC samples were distributed between the two groups (Fig. 2). The weighted UniFrac distance of the VVC, BV/VVC, and BV groups to the NC groups were significantly (Kruskal-Wallis One-way Analysis of Variance on Ranks, p<0.05) higher than the NC group. Moreover, the three disease groups were also significantly different (Fig. 2). Similar to the alpha-diversity results, the community structure of the VVC group was intermediate between the healthy and BV status.

**Figure 3:**
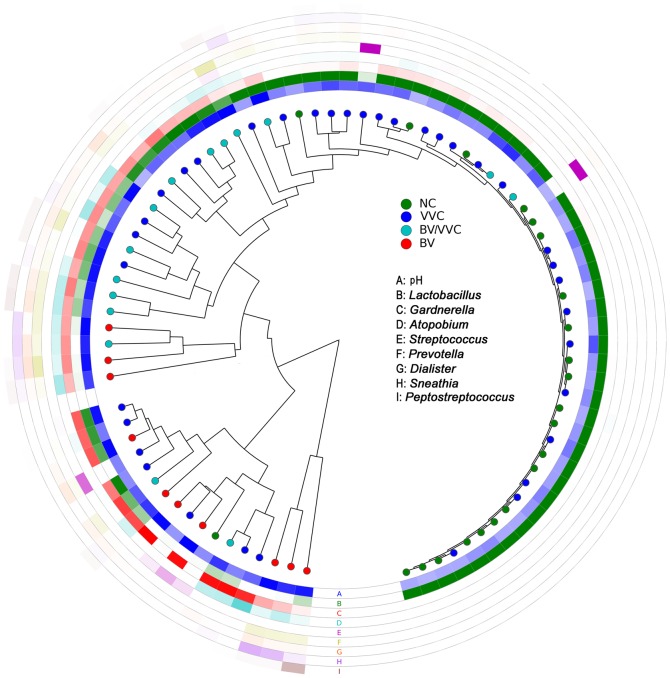
Clustering of the samples using weighted UniFrac distances. Each dot represents a sample, and each ring color represents a feature. A deeper ring color indicates that the value of that feature in that sample is larger.

The BV/VVC group exhibited a unique pattern of community structure, with clade grouping that differed from that of BV and NC (Fig. 3). The BV/VVC group showed an average of 46% *Lactobacillus*, visually higher than that of BV but lower than NC (Fig. 1). Although the BV/VVC group had a higher percentage of *Lactobacillus* than the BV group, bacteria from *Atopobium*, *Dialister*, *Gardnerella*, and *Prevotella* are also prevalent, similar to the BV microbiota. According to our results, the level of *Prevotella* was the highest in the BV/VVC group, with an LDA significance value of 4.0 by LEfSe (Fig. S1).

In contrast, the VVC group, representing the patients with VVC but not mixed with BV displayed no universal pattern for the vaginal microbiome community. Although the group, in total, has a higher species diversity and different community diversity than the NC group (as shown by the Shannon index and UniFrac distance in Fig. 2), the vaginal communities from VVC-only patients varied from a normal profile predominant with *Lactobacillus* to BV-like structures (Figs. 1 and 2). The variety of microbiota patterns within a single group could be observed by the span of the VVC samples in the PCoA results (Fig. 2). In our analyses, we observed that approximately 21 (54%) of the VVC samples were grouped with the ‘normal’ communities dominated by *Lactobacillus*. However, 7 VVC samples (18%) grouped with the BV group with abundant *Gardnerella* but very low levels of *Lactobacillus*. The remaining VVC samples were between these two extremes and clustered with the BV/VVC group (Fig. 3). We did not observe a significant correlation between the pH value and community diversity in the VVC patient group (Mantel test, R = 0.039, P = 0.73, 999 permutations).

### Microbiome after treatment

**Figure 4:**
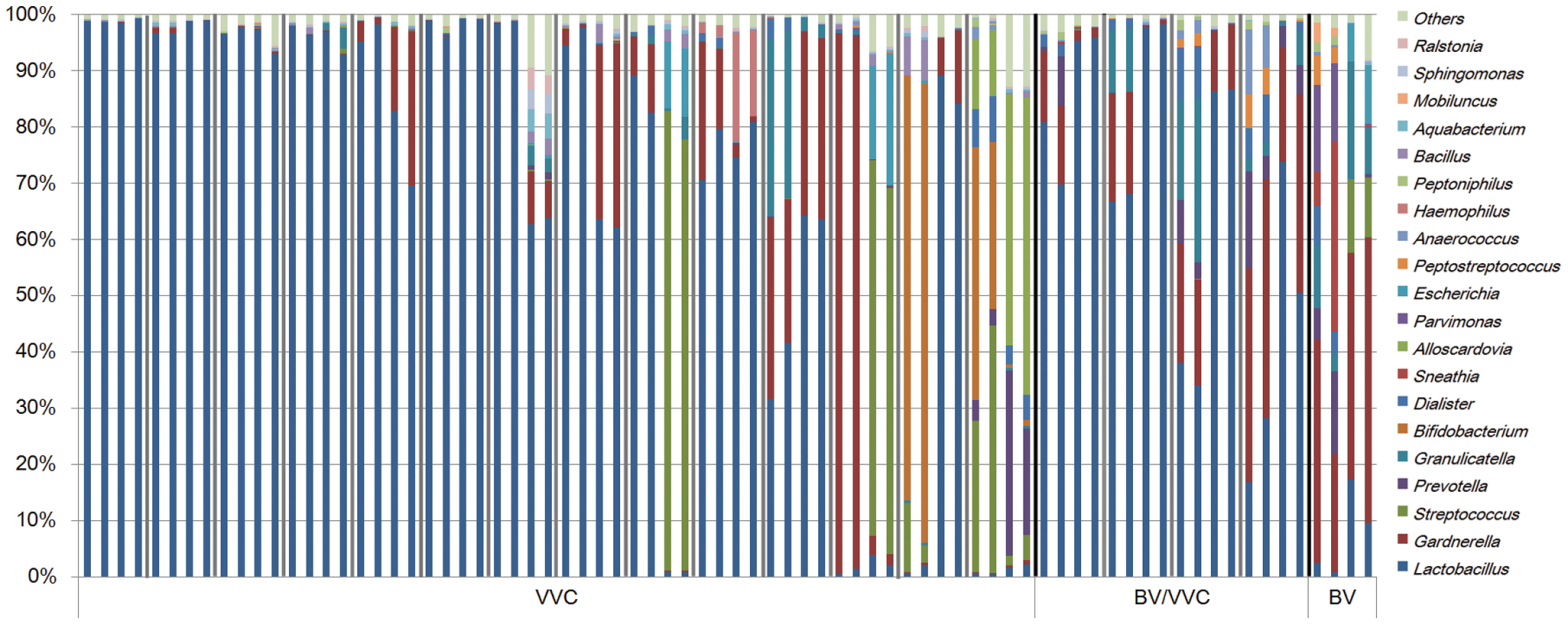
Comparison of community structures before and after treatment. Microbiomes from the same subject were grouped together. For each individual, the first two samples were those before treatment, and the last two were after treatment. Gray lines are used to separate different individuals, and black lines are used to separate different groups.

We successfully collected a total of 38 follow-up samples from 19 subjects consisting of 14 from the VVC-only, 4 from the BV/VVC, and 1 from the BV patients (Fig. 4). The VVC patients were treated with fluconazole; the BV/VVC patients were first treated with ornidazole and then fluconazole. The follow-up samples were collected at 9 to 16 days after the treatment when the patients revisited the hospital. Although all of the follow-up patients recovered with improved symptoms, many did not exhibit the expected healthy vaginal microbiomes (Fig. 4). For the VVC patients with initially normal vaginal microbiomes, three of the eight patients showed even higher levels of *Gardnerella* than before treatment. In comparison, the two individuals with unusual microbiomes recovered a *Lactobacillus*-abundant microbiome following the treatment. Interestingly, we observed two that VVC patients initially with an intermediate vaginal microbiota recovered to a *Streptococcus*-dominated vaginal microbiome that was similar to the unusual microbiomes found in some asymptomatic healthy women [3], [26], [29]. In comparison, all four samples with mixed BV/VVC showed increased *Lactobacillus* after treatment (Fig. 4).

## Discussion

The present study revealed for the first time the diverse patterns of the vaginal microbiome in reproductive-age women with VVC. No single profile can be used to describe the vaginal microbiome of all VVC patients. In comparison, our results for the vaginal microbiome of healthy women in China typically show a *Lactobacillus* predominance, with a pH lower than 4.5, which is similar to data reported for Asian women in America [3], [30], whereas the BV microbiomes generally exhibited a high diversity, with abundant *Gardnerella* and very low *Lactobacillus* [26]–[28]. Although studies involving the longitudinal sampling of healthy women show vaginal microbiome fluctuations from a *Lactobacillus*-dominated status to another structure, most of the snapshot profiles follow the pattern of *Lactobacillus* predominance [26].

The VVC samples can be divided into two major groups: one with mixed infection of BV and one with VVC only. Our results revealed a unique vaginal microbiota community structure in the patients with a mixed BV and VVC infection (Fig. 1 and Fig. 2), and it is interesting that the microbiome of the BV/VVC group is markedly different from the BV group (Figs. 1 and 2). The unique microbiome structure of the BV/VVC group suggests that the presence of candidiasis might alter the structure of an unhealthy microbiome (the BV microbiome). All the BV/VVC microbiomes showed abundant *Lactobacillus*, in contrast to the depletion of this genus in BV patients, indicating that *Candida* infection created an environment that was friendly to the growth of *Lactobacillus*. Another possibility is that both strains were simultaneously promoted by some environmental factor, such as estrogen [2]. Interestingly, all the follow-up samples in the BV/VVC and BV groups showed an increased abundance of *Lactobacillus*, indicating that treatment with antibiotics might be useful for the recovery of a healthy vaginal microbiome.

The VVC-only samples showed a wide variety of community structure. The data suggested that all the previous studies regarding the vaginal microbial communities of VVC were partly correct, considering the traditional cultivation or molecular fingerprinting methods that were used. Historically, mixed results have been reported for the vaginal microbiomes of VVC patients [9], [11]. Many studies have found that *Lactobacillus* spp. were still abundant in VVC and that there was no significant difference between the vaginal microbiomes of VVC patients and healthy controls [11]. Our study found that the vaginal microbiomes from most of the VVC subjects had abundant *Lactobacillus* strains, which supports the isolation of *Lactobacillus* from VVC patients in many previous reports [15]. *Gardnerella* was found in a large proportion of the VVC patients in our study as well, and the microbiome diversity was increased in these groups, consistent with the increased Nugent score in the literature [13], [15]. At present, we are unable to extrapolate any reason or meaning for these different types of microbiomes in VVC patients. It is possible that the variations merely reflect the changing patterns at different infection stages. The different patterns found in the VVC microbiomes appear to have no correlation with pH, as there were no significant differences in the pH values among the different VVC subgroups. Furthermore, because all our VVC patients presented such symptoms as discharge, “cottage cheese” discharge, vulval pruritus, and a burning sensation, the different patterns do not appear to be indicative of clinical symptoms. The present findings suggested that the complex vaginal microbiome community profiles of VVC can only be revealed by high-throughput methods with NGS techniques; indeed, the traditionally used methods might be the reason for the inconsistent results in previous studies.

Treatment, however, did alter the bacterial community structure, indicating that treatment of *Candida* infection affects the vaginal microbiome. Because of the relatively small follow-up sample, we could not determine consistent changes resulting from the treatments. However, we were surprised to find that many of the VVC patient microbiomes after treatment were similar to the unusual vaginal microbiomes of apparently healthy women, indicating that the unusual vaginal microbiomes might constitute the transition state between disease and health, particularly because there are many women with asymptomatic *Candida* infections [9].

Our study only addressed a portion of the complex vaginal microbiomes of women with VVC. To understand the interactions of fungi and bacteria within the human vagina and to personalize the treatment of vaginal infectious diseases, more studies using high-throughput sequencing techniques with longitudinal samples before and after treatment are warranted.

## Supporting Information

Figure S1 **Discriminative taxa determined by LEfSe.** Taxa with LDA values greater than two are displayed.
(PDF)
