# Supplementary figures and images for: Diverse Vaginal Microbiomes in Reproductive-Age Women with Vulvovaginal Candidiasis
Source: PLoS One. 2013 Nov 12;8(11):e79812. doi: 10.1371/journal.pone.0079812 (PMC3827160; doi:10.1371/journal.pone.0079812)

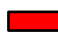 BV

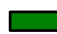 BV.VVC

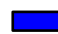 NC
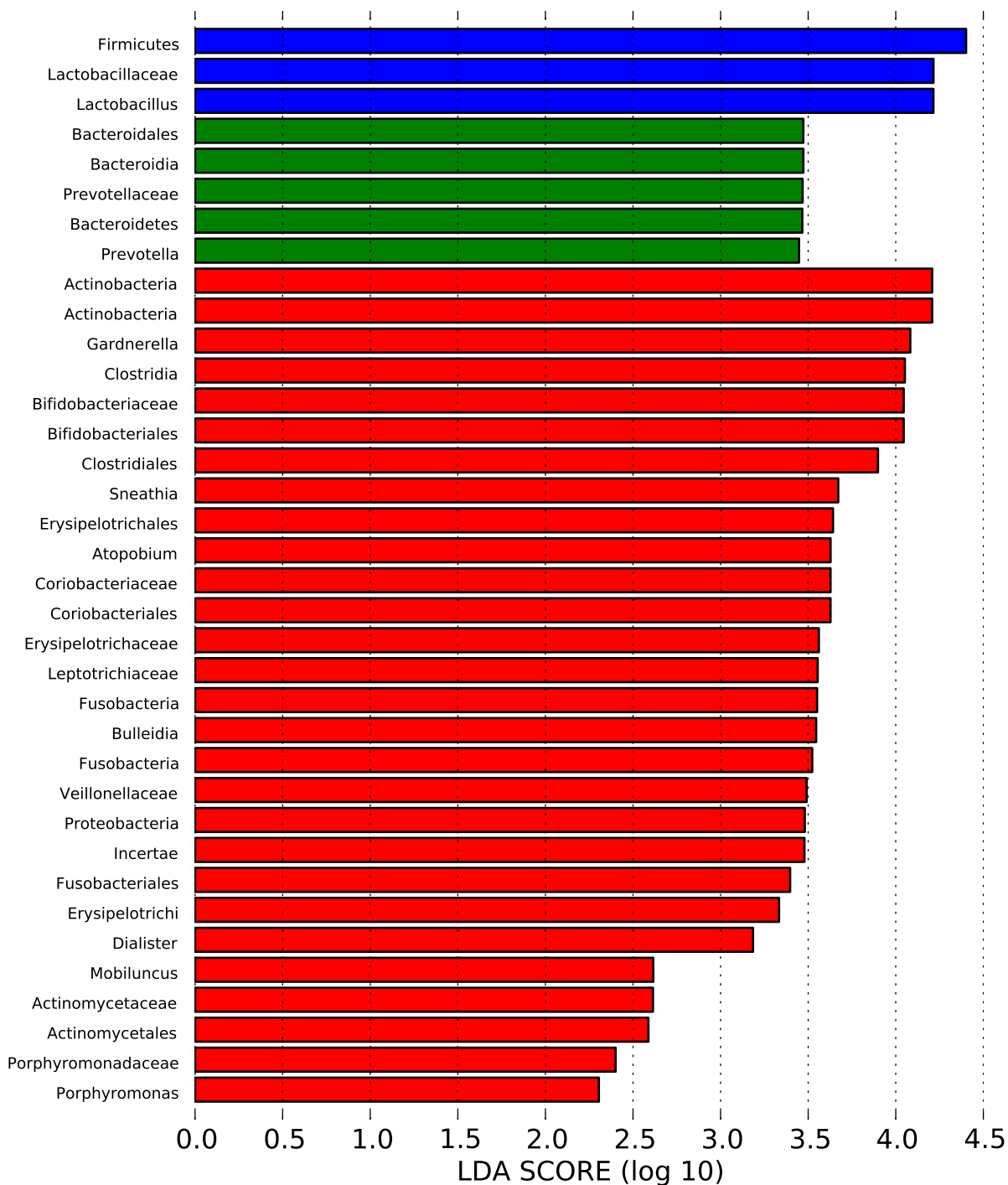

Supplement: Figure S1 — Discriminative taxa determined by LEfSe. Taxa with LDA values greater than two are displayed. (PDF) [file pone.0079812.s001.pdf]
